# Prostate tumours from an Asian population: examination of bax, bcl-2, p53 and *ras* and identification of bax as prognostic marker

**DOI:** 10.1054/bjoc.2000.1355

**Published:** 2000-08-17

**Authors:** S-J Chia, W-Y Tang, J Elnatan, W-M Yap, H-S Goh, D R Smith

**Affiliations:** Department of General Surgery, Molecular Biology Laboratory, Department of Pathology, Tan Tock Seng Hospital, Moulmein Road, 308433, Singapore

**Keywords:** immunohistochemistry, mutation, bax, bcl-2, p53, survival

## Abstract

Molecular studies have suggested that ethnicity may play a significant role in prostate tumorigenesis, but no information exists for groups other than Caucasian or Japanese patients. We examined 62 archival samples of prostate tumours from Asians of non-Japanese origin for the over-expression of p53, for the possible presence of mutated *ras* genes, for the overexpression of the bcl-2 and bax proteins, as well as directly for the presence of apoptotic cells by the TUNEL methodology. Gene lesions of both *ras*(0%) and p53 (3%) were rare. While bcl-2 expression was not observed in any sample, bax expression was noted in 76% of samples and was associated with a significantly worse patient prognosis both overall (*P*< 0.005) and specifically in Chinese patients (*P*< 0.02). Apoptotic cells were found in 61% of samples, and were significantly associated with the presence of bax expression (*P*= 0.002), but not patient survival. These results suggest that prostate tumours from non-Japanese Asians are genetically distinct from prostate tumour found in both Japanese and Caucasian patients, and that treatment modalities may need to be tailored for specific population groups. © 2000 Cancer Research Campaign


*British Journal of Cancer* (2000) **83**(6), 761–768
© 2000 Cancer Research Campaign
doi: 10.1054/ bjoc.2000.1355, available online at http://www.idealibrary.com on IDEAL®

### Prostate tumours from an Asian population: examination of bax, bcl-2, p53 and *ras* and identification of bax as a prognostic marker

**S-J Chia[1], W-Y Tang[2], J Elnatan[2], W-M Yap[3], H-S Goh[2] and DR Smith[2]**

[1]Department of General Surgery; [2]Molecular Biology Laboratory; [3]Department of Pathology, Tan Tock Seng Hospital, Moulmein Road, Singapore 308433

**Summary** Molecular studies have suggested that ethnicity may play a significant role in prostate tumorigenesis, but no information exists for groups other than Caucasian or Japanese patients. We examined 62 archival samples of prostate tumours from Asians of non-Japanese origin for the over-expression of p53, for the possible presence of mutated *ras* genes, for the overexpression of the bcl-2 and bax proteins, as well as directly for the presence of apoptotic cells by the TUNEL methodology. Gene lesions of both *ras* (0%) and p53 (3%) were rare. While bcl-2 expression was not observed in any sample, bax expression was noted in 76% of samples and was associated with a significantly worse patient prognosis both overall ($P < 0.005$) and specifically in Chinese patients ($P < 0.02$). Apoptotic cells were found in 61% of samples, and were significantly associated with the presence of bax expression ($P = 0.002$), but not patient survival. These results suggest that prostate tumours from non-Japanese Asians are genetically distinct from prostate tumour found in both Japanese and Caucasian patients, and that treatment modalities may need to be tailored for specific population groups. © 2000 Cancer Research Campaign

**Keywords:** immunohistochemistry; mutation; bax; bcl-2; p53; survival

Prostate cancer is currently the sixth most common cancer in Singapore (Chia et al, 1996). It has however been showing an average annual increase in incidence of some 4.6% over the last 25 years (Chia et al, 1996) and coupled with an ageing population (Goh, 1997), prostate cancer is expected to become a significant source of cancer-related mortality in Singapore. Prostate cancer is a heterogeneous disease, and as such presents significant problems in prognostication, despite a range of clinical and pathological markers of tumour aggressiveness. As such, attention has focused on understanding the underlying genetic events in attempts to determine appropriate clinicopathologic markers of tumour behaviour, as well as to aid in the development of novel treatment strategies.

The most commonly altered gene in a wide variety of tumours is the p53 tumour suppressor gene (Greenblatt et al, 1994) and in western population considerable data has shown that lesions of the p53 tumour suppressor gene are significantly associated with prostate cancer disease progression and a poorer patient prognosis, both with respect to overexpression of the protein (Thomas et al, 1993; Bauer et al, 1995; 1996; Shurbaji et al, 1995; Kuczyk et al, 1998) and mutation of the gene (Effert et al, 1992; Eastham et al, 1995; Stapleton et al, 1997). However, data on Japanese patients (Uchida et al, 1993; Watanabe et al, 1994) has shown that p53 overexpression and mutation occur in prostate tumour with a considerably lower frequency than is found in Western or Caucasian populations.

However, the p53 tumour suppressor gene is implicated in a wide range of cellular activities including DNA replication and regulation of the cell cycle. In particular the p53 tumour suppressor gene is involved in regulating some apoptotic pathways (Deppert, 1994) and it is possible that lesions elsewhere in the regulation of apoptosis are a predominant factor in prostate tumorigenesis in Asian populations. The p53 tumour suppressor gene is believed to control apoptosis by regulating the expression of the apoptosis associated genes bcl-2 and bax (Miyashita et al, 1994; Miyashita and Reed, 1995) and it is possible that alterations in the expression of one of these genes may result in a tumorigenic pathway the equivalent of a lesion in the p53 gene that is found in Western populations.

In addition to data showing discordant rates of p53 involvement in prostate tumours from Western and Japanese cohorts, further studies have also indicated sharply discordant rates of *ras* mutation. In particular studies on Japanese patients have shown rates of *ras* mutation of up to 25% (Anwar et al, 1992; Shiraishi et al, 1998), while studies on Caucasian patients note that this gene is rarely if ever mutated (Gumerlock et al, 1991; Moul et al, 1992).

In the absence of data on prostate tumorigenesis in population cohorts other than Caucasian or Japanese, we therefore undertook to examine archival specimens of prostate tumours from Singapore for a range of markers, including the expression of p53, bax and bcl-2, and the mutation of the *ras* family of genes. At the same time we also examined the samples directly for the presence of apoptotic cells using the terminal deoxynucleotide transferase (TdT)-mediated nick end labelling or TUNEL methodology (Gavrieli et al, 1992).

### MATERIALS AND METHODS

### Patients and samples

Samples used in this study were prostatic archival paraffin blocks obtained after transurethral resection of the prostate (TURP) from

*Received 29 November 1999*
*Revised 29 March 2000*
*Accepted 8 May 2000*

*Correspondence to:* DR Smith patients admitted to the Department of General Surgery, Tan Tock Seng Hospital between 1993 and 1998. No initial chemotherapy, radiotherapy or hormonal therapy was given before tumour excision. All samples were included only after histopathological diagnosis and tumours were graded according to the Gleason system (Gleason, 1977). All patients were classified according to ethnic group as given on their National Registration Identity Certificates, and comprised 50 Chinese, eight Malay and four Indian males. Mean age of patients was 75.19 ± 8.59 (range 45–91) years. All original haematoxylin and eosin slides were reviewed prior to selection of blocks containing the largest tumour volume. Slides were graded for gene expression by a single pathologist. At the end of the study period 29 (47%) patients had died of their disease. Mean follow-up was 28.66 ± 20.12 (range 1–77) months. Information on patient survival was obtained from the Singapore Cancer Registry and hospital clinical records. Patients that had died of causes other than their disease were censored from the survival analysis at the time of death. Lymph-node status and the presence of distant metastatic lesion were determined by CT pelvic scan and bone scan respectively. Fifty-eight percent of patients (36/62) underwent bilateral subcapsular orchidectomy. Fourteen of the patients in this cohort refused further investigation or treatment after surgery.

### Immunohistochemistry

Immunoreactive p53, bcl-2 and bax were detected by the labeled streptavidin–biotin method (Hsu et al, 1981). Several contiguous 10 μM sections were mounted on poly-L-Lysine treated slides, dewaxed in xylene and rehydrated in successive immersion in absolute ethanol, 70% ethanol and distilled water. One section was stained with haematoxylin and eosin. The remaining sections were extensively rinsed in phosphate buffered saline (PBS) and endogenous peroxidases were quenched in 3% hydrogen peroxide for 5 min. After washing in PBS slides were then incubated with blocking reagent for 20 min (Dako LSAB Kit, Dako Carpinteria, CA, USA). Incubation with primary antibody followed rinsing three times with PBS. Incubation was undertaken at 4°C overnight with either a 1:50 dilution of anti-p53 monoclonal antibody DO-7 (Dako), a 1:100 dilution of anti-bcl-2 polyclonal antibody Ab-2 (Oncogene Research Products, Cambridge, MA, USA) or a 1:100 dilution of anti-bax polyclonal antibody Ab-1 (Oncogene Research Products). Following three rinses with PBS, slides were then incubated with linking antibody (Dako) for 10 min, followed by 10 min with streptavidin–horseradish–peroxidase diluted as recommended by the manufacturer and then incubated for 8 min with the chromogen 3,3′-diaminobenzidine tetrahydrochloride (DAB; Dako). After each incubation samples were rinsed three times with PBS. Samples were counterstained with haemotoxylin for 1 min and nuclei blued in water. Slides were then dehydrated and mounted. Specificity of antibody binding was confirmed by undertaking, in parallel appropriate negative (no antibody) and positive controls. Positive controls included a fresh, flash-frozen colorectal adenocarcinoma previously characterized as strongly over-expressing p53 (Smith et al, 1996), as well as fresh, flash-frozen colorectal mucosae for bax and bcl-2. Pattern of expression for bax and bcl-2 control specimens conformed to established profiles of expression for these genes (Ogura et al, 1999). Analysis was undertaken in duplicate on successive occasions. All specimens were examined across the whole section by light microscopy, and were deemed positive if there was evidence of focal positivity or greater.

### TUNEL analysis

TUNEL analysis (Gavrieli et al, 1992) was undertaken with the Cell Death Kit (Boehringer Mannheim GmbH, Penzburg, Germany) essentially as recommended by the manufacturer. Briefly, samples were dewaxed in two changes of xylene followed by sequential rehydration in two changes of ethanol (absolute and 70%) followed by distilled water. Tissue sections were incubated with 25 μg $ml^{-1}$ Proteinase K in 10 mM Tris-HCl, pH 7.4 for 15 min at room temperature. Following rinsing in PBS, exogenous peroxidases were quenched in 3% hydrogen peroxide for 5 min. After rinsing in PBS, slides were incubated in blocking solution for 30 min at room temperature. Slides were incubated with 100 μl of the TUNEL reaction mixture for 60 min at room temperature in a humidified atmosphere. Following rinsing in PBS slides were incubated with 100 μl converter-peroxidase solution, pre-diluted 1:5 in blocking solution and incubated for a further 30 min at room temperature. After rinsing in PBS samples were incubated with 1 mg $ml^{-1}$ DAB substrate and incubated for 8 min at room temperature. Slides were subsequently washed, counterstained with haemotoxylin and mounted. Specificity of TUNEL reactivity was confirmed by undertaking in parallel appropriate negative (no antibody) and positive (fresh, flash-frozen colorectal mucosae) controls. Pattern of TUNEL expression was as reported by others (Moss et al, 1996).

### *Ras* analysis

Genomic DNA was prepared from one 10 μm thick section of paraffin-embedded tissue from each tumour. Tissue section was placed in a 1.5 ml centrifuge tube together with 1 ml of xylene and incubated on a tube rotator for 30 min at room temperature and contents pelleted by centrifugation. Two further resuspension in xylene and pelleting steps were undertaken. The pellet was washed twice with absolute ethanol and air-dried. Pellet was resuspended in 100 μl $H_2O$ containing 20 μg Proteinase K (Boehringer Mannheim) and 5% SDS and incubated for 3 days at 50°C. Proteinase K was inactivated by heating to 90°C and debris pelleted by centrifugation. The supernatant was extracted twice with an equal volume of phenol and once with chloroform. DNA was precipitated by adding 2.5 volumes ice-cold ethanol and sodium acetate to 0.3 molar and incubating at –80°C for 15 min followed by pelleting by centrifugation. DNA was resuspended in sterile distilled water.

### *c-Ki*-ras

A 176 bp fragment of exon 1 of the c-Ki-*ras* gene was amplified from all DNA samples using custom synthesized oligonucleotides: 5′-CATGTTCTAATATAGTCACA-3′ (forward); and 5′-AACAAGATTTACCTCTATTG-3′ (reverse) essentially as described previously (Elnatan, 1996*a*; 1996*b*).

### *H*-ras

A 145 bp fragment of exon 1 of the H-*ras* gene was amplified from all DNA samples using the custom synthesized oligonucleotides: 5′-GGCAGGAGACCCTGTAGGAG-3′ (forward); and 5′-GTATTCGTCCACAAAATGGTTCT-3′ (reverse): Following an

 © 2000 Cancer Research Campaign initial denaturation at 94°C for 4 min, samples were amplified by 40 cycles of 94°C for 1 min (denature), 50°C for 2 min (anneal) and 72°C for 3 min (extend).

### *N*-ras

A 109 bp fragment of exon 1 of the N-*ras* gene was amplified from all samples using the custom synthesized oligonucleotides: 5′-GACTGAGTACAAACTGGTGG-3′ (forward); and 5′-CTC-TATGGTGGGATCATATT-3′ (reverse). Following an initial denaturation at 94°C, samples were subjected to 40 cycles of 94°C for 1 min (denature), 50°C for 2 min (anneal) and 72°C for 3 min (extend).

### Single-stranded conformational polymorphisms (SSCP)

All *ras* PCR products were analysed by single-stranded conformational polymorphism analysis (Orita et al, 1989). Briefly, *ras* PCR products were denatured at 95°C and loaded onto a 15% (K-*ras* and N-*ras*) or 28% (N-*ras*) 29:1 acrylamide:bisacrylamide polyacrylamide gel containing 5% glycerol Mighty Small™ (Hoefer, Amersham Pharmacia Biotech, Uppsala, Sweden). Samples were electrophoresed at 200 volts for 3.5 h. After electrophoresis, DNA bands were developed by silver staining using the Hoefer Automated Gel Stainer (Hoefer, Amersham Pharmacia Biotech) and the protocol of Soong and Iacopetta (1997).

### p53 mutation analysis

Samples positive for p53 overexpression were subjected to exon-specific PCR for exons 4–9 of the p53 gene followed by SSCP analysis essentially as described above. Aberrantly migrating bands were excised from the gel matrix after silver staining using the protocol of Soong and Iacopetta (1997), and re-amplified using the same exon-specific primers. The amplification products were then purified by agarose gel electrophoresis and excised from the gel matrix, after which templates were purified using the Qiaex PCR Purification Kit (Qiagen, Hilden, Germany). DNA sequencing reactions were performed using the Big Dye Termination reaction kit (PE Applied Biosystems, Foster City, CA, USA) on approximately 60–90 ng of template from above. Analysis was undertaken on an automated ABI 377-18 DNA Sequencer (PE Applied Biosystems).

### Statistical analysis

Two by two tables were analysed by the $\chi^2$ test. Univariate survival analysis was carried out using the method of Kaplan–Meier and differences between survival curves were compared using the log-rank test. All tests were two-tailed and statistical significance was assumed when $P < 0.05$. Analyses were carried out using the SPSS software package (Chicago, IL, USA).

### RESULTS

Sixty-two formalin fixed, paraffin embedded specimens of prostatic cancer from Singaporean patients were analysed for the pattern of expression of p53, bcl-2 and bax, as well as for the presence of apoptotic cells using the TUNEL methodology (Table 1). At the same time samples were also examined for possible mutation of K-*ras*, H-*ras* and N-*ras*. For all samples one 5 µm section was stained with haematoxylin and eosin to confirm identity of the sample and to identify areas of necrosis prior to further analysis.

### p53 overexpression and mutation

Overexpression of the p53 protein using monoclonal antibody DO-7 was detected in tumours from two patients (3%). In both cases staining was nuclear (Figure 1). To determine if overexpression was the result of a point mutation of the p53 gene, we extracted DNA from further sections of these two samples and undertook exon-specific PCR for exons 4–9 of the p53 gene, and products were analysed by SSCP (Orita et al, 1989). Aberrantly migrating bands were observed for exon 7 for both samples after silver staining of the gel. The aberrant bands were excised from the gel and subjected to re-amplification with exon 7-specific primers. Products were analysed by DNA sequencing and analysis on an ABI automated sequencer. DNA sequence analysis (Figure 1) determined that the lesions in these samples were a G to C transversion resulting in the substitution of a glycine with an alanine at codon 245 for one patient and a G to A transition resulting in the substitution of an Arginine with a Histidine at codon 237 for the second patient.

### Analysis of *ras* genes

Genomic DNA was prepared from one 10 µm section from all samples. This was used as a template to amplify portions of the K-*ras*, N-*ras* and H-*ras* exon 1. These samples were screened by Single Stranded Conformational Polymorphisms (Figure 2), a technique we have previously shown to be sensitive to the presence of *ras* mutations in colorectal adenocarcinomas (Eluatan, 1996*a*; 1996*b*). Of the 186 PCR products analysed, a single sample with aberrantly migrating bands was detected. This was in the H-*ras* gene of one patient. The aberrantly migrating bands were excised from the gel matrix after silver-staining, re-amplified with H-*ras* exon 1-specific primers and analysed by DNA sequencing and analysis on an Automated ABI 377 DNA Sequencer. The mutation was determined to be a G to T transversion at position –10 with respect to the A of the translation start site ATG.

### Bax and bcl-2 expression

Analysis of bax protein expression was undertaken with a monoclonal antibody directed against human bax (Ab-1). Evidence of bax protein expression was detected in the neoplastic cells in 76% (47/62) of samples (Figure 3, Table 1). Consistent with the known location of the bax protein (Wolter et al, 1997), staining was cellular. Bax staining was heterogeneous with some samples showing extensive staining across the entire section, while others showed a relatively restricted distribution to one or a few regions of the sample. In eight cases normal and/or hyperplastic cells were also present in the section, all of which were positive for bax expression. In five of these cases the neoplastic cells were also positive for bax expression, while in three cases normal/hyperplastic tissues were positive while the tumour mass was negative for bax expression. No case of bcl-2 immunostaining was observed (Figure 3), although staining was appropriately noted in positive control samples (data not shown). Bcl-2 immunoreactivity was also not noted in either normal or hyperplastic tissues. Univariate survival analysis was undertaken with patients stratified according to bax expression status. Patients who expressed

© 2000 Cancer Research Campaign

**Table 1** Summary of results from 62 patients

| Sample /Race[a] | Age | Gleason score | Lymph node[b] | Stage | Distant metastases[c] | POT[d] | p53 | bax | TUNEL | Follow-up | Status |
|---|---|---|---|---|---|---|---|---|---|---|---|
| 1/C | 73 | 4+5 | Y | D2 | Y | Y | N | N | N | 10 | Dead |
| 2/C | 78 | 4+4 | Y | C1 | N | Y | N | N | N | 77 | Alive |
| 3/C | 74 | 5+4 | Y | D2 | Y | Y | N | N | N | 25 | Dead |
| 4/C | 81 | 4+5 | N | B | N | Y | N | Y | N | 27 | Dead |
| 5/C | 61 | 5+2 | Y | D2 | Y | Y | N | Y | Y | 14 | Dead |
| 6/C | 87 | 2+1 | N | A2 | ND | N | N | Y | N | 10 | Dead |
| 7/C | 75 | 3+2 | N | ND | ND | Y | N | N | N | 72 | Alive |
| 8/C | 80 | 3+1 | Y | D | N | Y | N | Y | N | 23 | Dead |
| 9/C | 75 | 4+3 | ND | ND | ND | ND | N | Y | Y | 5 | Dead |
| 10/C | 63 | 4+3 | Y | D3 | Y | Y | N | Y | Y | 34 | Dead |
| 11/C | 68 | 4+3 | Y | D3 | Y | Y | N | Y | Y | 28 | Dead |
| 12/C | 75 | 3+2 | N | C1 | ND | N | N | Y | N | 23 | Dead |
| 13/C | 70 | 2+3 | N | B | ND | N | N | Y | Y | 24 | Dad |
| 14/C | 76 | 3+4 | N | B | N | Y | N | N | N | 69 | Alive |
| 15/C | 79 | 2+3 | ND | ND | ND | ND | N | N | N | 66 | Alive |
| 16/C | 67 | 5+5 | ND | ND | ND | Y | N | N | N | 2 | Alive |
| 17/C | 75 | 5+3 | Y | D3 | Y | Y | N | Y | Y | 1 | Dead |
| 18/C | 77 | 4+4 | ND | ND | ND | ND | N | Y | Y | 8 | Dead |
| 19/C | 78 | 5+4 | Y | D3 | Y | N | N | Y | N | 1 | Dead |
| 20/C | 90 | 2+3 | Y | D3 | Y | Y | N | Y | Y | 13 | Dead |
| 21/C | 63 | 3+3 | N | A1 | N | N | N | N | N | 58 | Alive |
| 22/C | 83 | 3+4 | Y | D2 | Y | Y | N | Y | Y | 49 | Dead |
| 23/C | 65 | 3+5 | Y | D1 | N | Y | N | Y | Y | 5 | Dead |
| 24/C | 71 | 3+4 | Y | D2 | Y | Y | N | Y | Y | 55 | Alive |
| 25/C | 63 | 4+3 | Y | D2 | Y | Y | N | Y | Y | 30 | Alive |
| 26/C | 81 | 5+2 | N | D2 | Y | Y | Y | Y | N | 24 | Alive |
| 27/C | 81 | 4+5 | Y | D2 | Y | Y | N | Y | Y | 12 | Dead |
| 28/C | 84 | 4+3 | Y | D2 | Y | Y | N | Y | Y | 23 | Alive |
| 29/C | 69 | 3+2 | N | C1 | N | N | N | Y | Y | 22 | Alive |
| 30/C | 84 | 3+4 | N | B | N | Y | N | Y | N | 21 | Alive |
| 31/C | 89 | 4+3 | Y | D2 | Y | Y | N | Y | Y | 5 | Dead |
| 32/C | 64 | 4+5 | Y | D1 | N | Y | N | Y | N | 53 | Alive |
| 33/C | 69 | 3+4 | N | A2 | N | Y | N | N | Y | 52 | Alive |
| 34/C | 88 | 5+4 | ND | ND | ND | ND | N | Y | N | 20 | Dead |
| 35/C | 79 | 3+4 | ND | D1 | ND | N | N | Y | Y | 47 | Alive |
| 36/C | 82 | 4+5 | ND | ND | ND | ND | N | Y | Y | 1 | Dead |
| 37/C | 78 | 4+3 | N | A2 | N | Y | N | Y | N | 14 | Dead |
| 38/C | 74 | 1+2 | N | A2 | N | Y | N | N | Y | 43 | Alive |
| 39/C | 69 | 3+4 | N | D2 | Y | Y | N | Y | Y | 43 | Alive |
| 40/C | 68 | 5+5 | N | A1 | N | Y | N | N | Y | 39 | Alive |
| 41/C | 81 | 4+3 | Y | D2 | Y | Y | N | Y | Y | 37 | Alive |
| 42/C | 91 | 3+5 | Y | D2 | Y | Y | N | Y | Y | 33 | Alive |
| 43/C | 86 | 3+4 | N | A2 | N | N | N | Y | Y | 32 | Alive |
| 44/C | 78 | 4+5 | N | A2 | N | Y | N | Y | Y | 13 | Dead |
| 45/C | 79 | 4+5 | Y | D2 | Y | Y | N | Y | Y | 30 | Alive |
| 46/C | 68 | 4+5 | Y | D2 | Y | Y | N | Y | Y | 29 | Alive |
| 47/C | 83 | 4+5 | N | D2 | Y | Y | N | Y | Y | 28 | Alive |
| 48/C | 76 | 4+3 | Y | D3 | Y | Y | N | Y | Y | 28 | Alive |
| 49/C | 78 | 4+5 | N | A1 | N | Y | N | N | N | 27 | Alive |
| 50/C | 64 | 3+4 | N | D2 | Y | Y | N | Y | Y | 25 | Alive |
| 51/M | 72 | 4+2 | Y | D2 | Y | Y | N | N | N | 77 | Alive |
| 52/M | 82 | 5+4 | N | C1 | N | Y | N | Y | Y | 15 | Dead |
| 53/M | 45 | 4+4 | ND | D | ND | ND | N | Y | N | 16 | Dead |
| 54/M | 79 | 4+3 | Y | C1 | ND | Y | N | N | Y | 65 | Alive |
| 55/M | 67 | 4+5 | Y | D2 | Y | N | N | Y | Y | 13 | Dead |
| 56/M | 87 | 4+4 | Y | D1 | Y | Y | N | Y | Y | 51 | Alive |
| 57/M | 79 | 5+4 | N | A2 | N | Y | N | Y | Y | 4 | Dead |
| 58/M | 66 | 4+5 | N | D2 | Y | Y | N | Y | N | 13 | Dead |
| 59/I | 80 | 2+3 | N | A2 | N | N | N | N | N | 27 | Alive |
| 60/I | 66 | 3+4 | Y | D1 | N | Y | Y | Y | Y | 33 | Alive |
| 61/I | 69 | 3+4 | ND | A1 | ND | Y | N | Y | Y | 10 | Dead |
| 62/I | 80 | 4+3 | N | A2 | N | Y | N | Y | N | 23 | Alive |

[a]Race: C = Chinese, M = Malay, I = Indian; [b]Lymph node status: Y = involved, N = not involved; [c]Distant metastases: Y = distant metastasis, N = no distant metastasis; [d]POT (administration of post-operative therapy): Y = yes, N = no

*© 2000 Cancer Research Campaign*

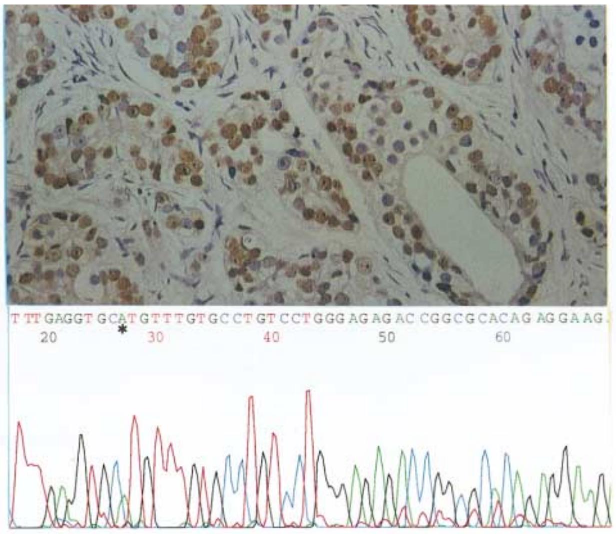

**Figure 1** Immunohistochemical and sequence analysis of a prostatic tumour specimen. (Top) Section of an archival prostatic tumour from an (Indian) Singaporean after staining with monoclonal antibody DO7 (magnification × 200). (Bottom) Partial DNA sequence of exon 7 of the p53 gene from the same patient as above after analysis on an Automated ABI DNA Sequencer. The mutation (G to A) is indicated (*)

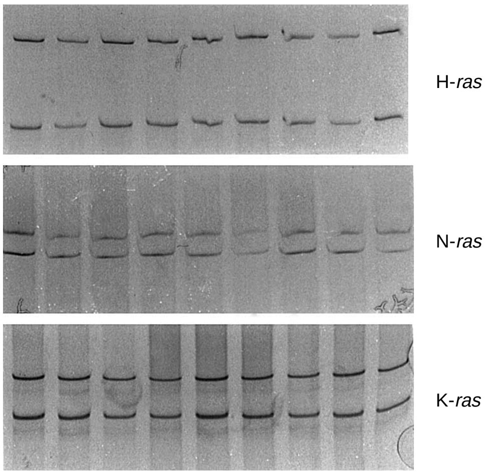

**Figure 2** Single stranded conformational polymorphism analysis of exon 1 of the H-*ras*, N-*ras* and K-*ras* genes of nine prostatic tumours. PCR samples are denatured and run on appropriate polyacrylamide gels. After electrophoresis samples are detected by silver-staining

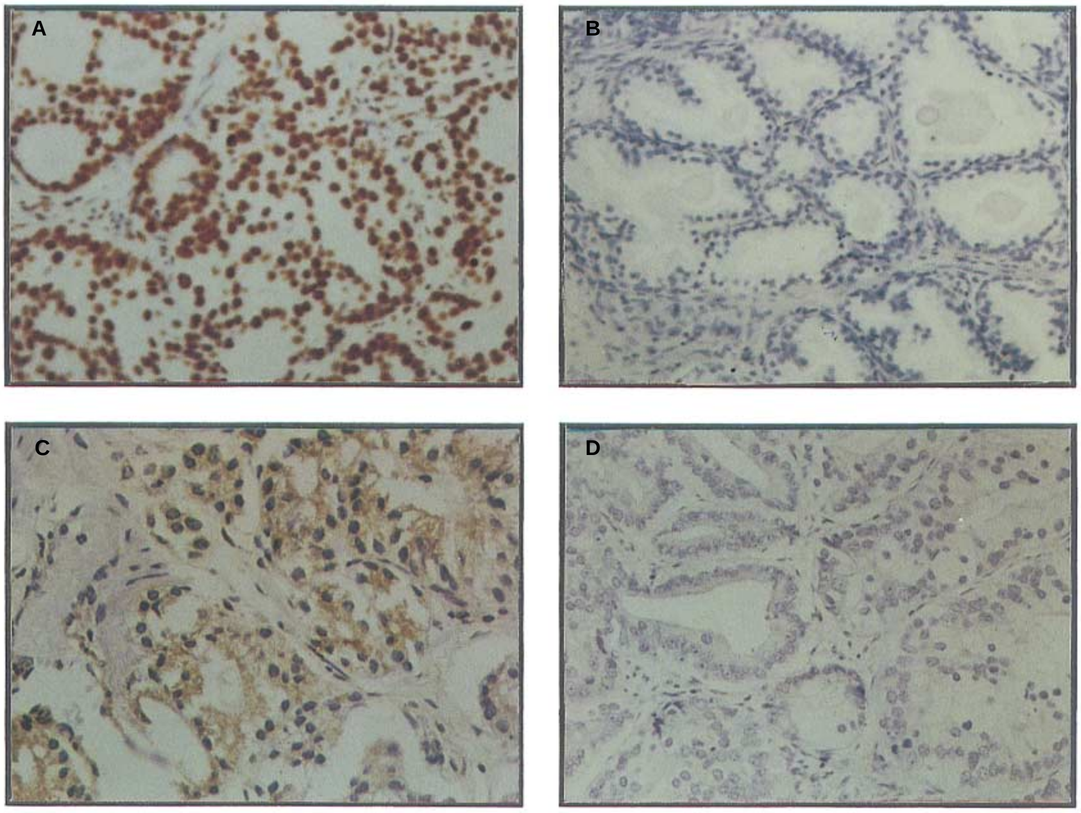

**Figure 3** Immunohistochemical analysis of prostate carcinoma specimens. Formalin-fixed, paraffin-embedded prostatic carcinoma sections positive (**A**) and negative (**B**) for apoptotic cells (TUNEL analysis) and positive for bax (**C**) and negative for bcl-2 (**D**). **A**, **C** and **D** are from one patient. Original magnification × 100

*© 2000 Cancer Research Campaign*

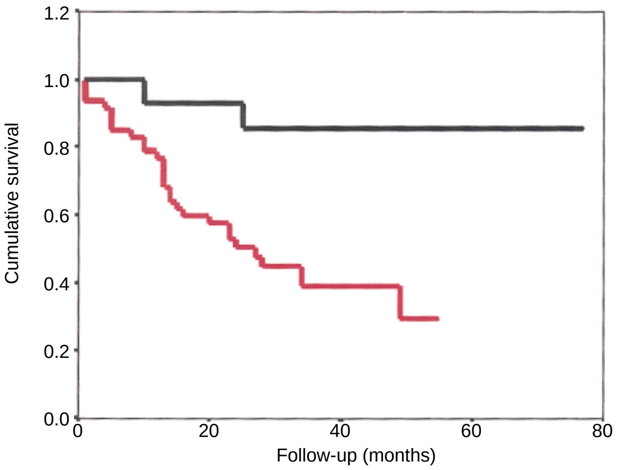

**Figure 4** Survival analysis for 50 Chinese prostate tumour patients stratified according to bax protein expression status. Black line: no bax expression observed; red line: bax expression observed. The two curves are significantly different ($P < 0.02$, log rank analysis)

bax were shown to have a significantly poorer long-term survival than those patients who did not express bax, both in the whole patient cohort ($P < 0.005$) and in a sub-cohort of 50 patients of Chinese origin ($P = 0.02$, Figure 4). Fourteen of the patients in this cohort refused further investigation or treatment, and hence information on lymph-node status and metastatic lesions is only available for 48 of the patients. We attempted to undertake a multivariate analysis using this smaller cohort. However, none of the terms were entered into the model, although bax expression remained the variable with the highest level of significance ($P = 0.0535$).

### TUNEL analysis

Terminal deoxynucleotide transferase (TdT)-mediated nick end labeling staining was undertaken on all samples, and 61% (38/62) of samples evinced some positively staining cells, although only 46% showed evidence of heavy and/or extensive staining (Figure 3). The presence of apoptotic cells was positively associated with the presence of bax expression in both the whole patient cohort ($P = 0.002$, Fisher's Exact Test, Table 2), as well as in a sub-cohort of 50 patients of Chinese origin ($P = 0.005$, Fisher's Exact Test, Table 2). Univariate survival analysis noted no association between survival and the presence or absence of apoptotic cells, in either the whole cohort ($P = 0.8$) or the sub-cohort of Chinese patients ($P = 0.9$).

### DISCUSSION

In Western population cohorts activation of any member of the *ras* gene family is rare (Gumerlock et al, 1999; Moul et al, 1992), and it is believed that *ras* activation does not play a significant role in prostate tumorigenesis. In contrast, Anwar and colleagues (1992) have reported a rate of 24% of prostatic carcinomas from Japanese patients showing mutation of either H-*ras* (predominantly), N-*ras* or K-*ras*. While some studies on prostatic carcinomas from Japanese patients have reported somewhat lower rates of *ras* activation (Konishi et al, 1997), it is clear that unlike prostate cancers

**Table 2** Association of bax expression with presence of TUNEL positive cells. Figures in brackets are for Chinese patients only

| | TUNEL Negative | TUNEL Positive |
|---|---|---|
| Bax Negative | 11 (9) | 4 (3) |
| Bax Positive | 13 (10) | 34 (28) |

from Western cohorts, activation of *ras* genes is a significant event in prostate tumorigenesis in Japanese patients. The patients examined here are the first non-Caucasian, non-Japanese patients to be examined for *ras* mutation, and no case of *ras* mutation was observed. This is unlikely to be due to technical or sensitivity problems as we have previously extensively analysed *ras* mutations in colorectal adenocarcinomas (Elnatan, 1996*a*; 1996*b*), and here detected one base alteration in a prostate carcinoma from a Singaporean Indian patient. The position of the alteration, within the 5′-untranslated region makes it unlikely to have functional significance, and may indeed represent a polymorphic variant. The lack of somatic material makes it impossible to distinguish this change as tumour-specific. These results suggest that like prostate cancers from Western cohorts, *ras* mutation in non-Japanese Asian prostate cancers is unlikely to play a biologically significant role.

In cohorts from Western countries such as the USA, lesions of the p53 gene (such as overexpression of the protein or mutation of the gene) are found in up to 65% of prostate tumours (Theodorescu et al, 1997) and such lesions have been shown to be significantly associated with both tumour progression and a poorer patient prognosis (Effert et al, 1992; Thomas et al, 1993; Bauer et al, 1995; 1996; Eastham et al, 1995; Shurbaji et al, 1995; Stapleton et al, 1997; Kuczyk et al, 1998). In these cohorts, bcl-2 expression is elevated (Bauer et al, 1995) and bcl-2 expression has been found to be associated with tumour progression (Furuya et al, 1996; Krajewska et al, 1996; Johnson et al, 1998) and a poorer patient prognosis (Bauer et al, 1995). It would seem that in these patients, p53 mutation results in a release of the suppression of bcl-2, leading to loss of appropriate apoptosis, as well as an insensitivity to apoptotic stimuli (Huang et al, 1998; Johnson et al, 1998). The finding that bax is expressed in all prostate tumours (Krajewska et al, 1996; Johnson et al, 1998) would indicate that the mutated p53 retains the ability to activate the transcription of bax, a finding that is consistent with data that shows that the different functions of p53 (apoptosis and transcription) can be differently inactivated by non-sense mutations (Bissonette et al, 1997; Guillouf et al, 1998).

In Asian cohorts however, the involvement of p53 (overexpression and/or mutation of the gene) is significantly lower, ranging from 3% in this study to approximately 10% in studies from Japan (Uchida et al, 1993; Watanabe et al, 1994), and both p53 and bcl-2 overexpression have been shown not to have prognostic information in a cohort of Japanese patients (Masuda et al, 1998). In our cohort of patients we find no evidence of bcl-2 expression in the tumour mass. This result would be consistent with the low rate of p53 inactivation found in these tumours, with wild-type p53 being able to appropriately suppress bcl-2 expression.

While bax expression has been noted in all tumour samples from Caucasian cohorts (Krajewska et al, 1996; Johnson et al, 1998), we note bax expression in only some 76% of samples. Where bax is expressed, we note, as would be expected, a significant relationship with the presence of apoptotic cells. These results would tend to suggest that, in Asian cohorts, the apoptotic

© 2000 Cancer Research Campaign pathway, at least with respect to p53, bax and bcl-2, is relatively intact in the majority of tumours. Some 22% of tumours show no evidence of bax expression, and only 26% of these tumours show evidence of apoptotic cells. In these tumours therefore, the reduction in bax expression would perhaps be functionally equivalent to an increase in bcl-2 seen in Caucasian populations (Bauer et al, 1995; Furuya et al, 1996; Krajewska et al, 1996; Johnson et al, 1998).

However, perhaps most surprising is the association between the expression of bax and a poorer patient prognosis, a result found both in the whole cohort, and in the subcohort of Chinese patients. This result would suggest that the expression of bax is associated with a more aggressive disease, and as such it is possible that these tumours have cell populations that are turning over very rapidly, and therefore have a greater probability of acquiring additional genetic changes necessary for a more malignant phenotype. While in some tumours, particularly those with a high proportion of microsatellite instability (Thibodeau et al, 1993) such as hereditary non-polyposis coli (Yagi et al, 1998) and gastric cancer (Oliveira et al, 1998), the bax gene itself is a target of somatic mutation, in these prostate tumours that is unlikely as there is a strong relationship between bax expression and the presence of TUNEL positive cells, suggesting that the bax protein is functionally active.

The summation of results here, for p53, *ras*, bcl-2 and bax describe a prostate tumour that is genetically distinct from those tumours from either Caucasian or Japanese cohorts, which in turn are genetically distinct from each other. As such, prostate tumorigenesis may represent the clearest example of genetic background or ethnicity determining the pathway to tumorigenesis.

In the future, treatment options for prostate cancer are likely to draw significantly upon our understanding of prostate tumour biology and are likely to target specific genetic lesions (Bower and Waxman, 1997). Given that evidence to date suggests that Asian and Caucasian populations do not show similar routes to prostate tumorigenesis, this will have profound effects on the viability of novel anti-tumorigenic treatment regimes in Asian patients.

### ACKNOWLEDGEMENTS

This work was supported by the Shaw Foundation, Singapore and Research Grant RSCH/97/007 from Tan Tock Seng Hospital.

### REFERENCES

Anwar K, Nakakuki K, Shiraishi T, Naiki H, Yatani R and Inuzuka M (1992) Presence of *ras* oncogene mutations and human papillomavirus DNA in human prostate carcinomas. *Cancer Res* **52**: 5991–5996

Bauer JJ, Sesterhenn IA, Mostofi KF, McLeod DG, Srivastava S and Moul JW (1995) p53 nuclear protein expression is an independent prognostic marker in clinically localized prostate cancer patients undergoing radical prostatectomy. *Clin Cancer Res* **1**: 1295–1300

Bauer JJ, Sesterhenn IA, Mostofi FK, McLeod DG, Srivastava S and Moul JW (1996) Elevated levels of apoptosis regulator proteins p53 and bcl-2 are independent prognostic biomarkers in surgically treated clinically localized prostate cancer. *J Urol* **156**: 1511–1516

Bissonnette N, Wasylyk B and Hunting DJ (1997) The apoptotic and transcriptional transactivation activities of p53 can be dissociated. *Biochem Cell Biol* **75**: 351–358

Bower M and Waxman J (1997) Gene therapy for prostate cancer. *Semin Cancer Biol* **8**: 3–9

Chia KS, Lee HP, Seow A and Shanmugaratnam K (1996) *Trends in Cancer Incidence in Singapore 1968–1992*. Singapore Cancer Registry Report No 4

Deppert W (1994) The yin and yang of p53 in cellular proliferation. *Semin Cancer Biol* **5**: 187–202

Eastham JA, Stapleton AMF, Gousse AE, Timme TL, Yang G, Slawin KM, Wheeler TM, Scardino PT and Thompson TC (1995) Association of p53 mutations with metastatic prostate cancer. *Clin Cancer Res* **1**: 1111–1118

Effert PJ, Neubauer A, Walther PJ and Liu ET (1992) Alterations of the P53 gene are associated with the progression of a human prostate carcinoma. *J Urol* **147**: 789–793

Elnatan J, Goh HS and Smith DR (1996*a*) C-KI-*RAS* activation and the biological behaviour of proximal and distal colonic adenocarcinomas. *Eur J Cancer* **32A**: 491–497

Elnatan J, Goh H-S and Smith DR (1996*b*) A comparison of the detection of activated c-Ki-*ras* genes by mismatch specific oligonucleotide hybridisation analysis and enriched PCR in colorectal adenocarcinomas. *Int J Oncology* **8**: 139–143

Furuya Y, Krajewski S, Epstein JI, Reed JC and Isaacs JT (1996) Expression of bcl-2 and the progression of human and rodent prostatic cancers. *Clin Cancer Res* **2**: 389–398

Gavrieli Y, Sherman Y and Ben-Sasson SA (1992) Identification of programmed cell death in situ via specific labeling of nuclear DNA fragmentation. *J Cell Biol* **119**: 493–501

Gleason DF (1977) Histological grading and clinical staging of prostate carcinoma. In *Urologic Pathology: The Prostate*, pp 171–197. Lea and Febiger: Philadelphia

Goh LG (1997) Future health issues and delivery needs of the elderly. *Singapore Med J* **38**: 418–421

Greenblatt MS, Bennett WP, Hollstein M and Harris CC (1994) Mutations in the p53 tumor suppressor gene: clues to cancer etiology and molecular pathogenesis. *Cancer Res* **54**: 4855–4878

Guillouf C, Rosselli F, Sjin RT, Moustacchi E, Hoffman B and Liebermann DA (1998) Role of a mutant p53 protein in apoptosis: characterization of a function independent of transcriptional trans-activation. *Int J Oncol* **13**: 107–114

Gumerlock PH, Poonamallee UR, Meyers FJ and deVere White RW (1991) Activated *ras* alleles in human carcinoma of the prostate are rare. *Cancer Res* **51**: 1632–1637

Hsu S-M, Raine L and Fanger H (1981) Use of avidin-biotin-peroxidase complex (ABC) in immunoperoxidase techniques: a comparison between ABC and unlabeled antibody (PAP) procedures. *J Histochem Cytochem* **29**: 577–580

Huang A, Gandour-Edwards R, Rosenthal SA, Siders DB, Deitch AD and White RW (1998) p53 and bcl-2 immunohistochemical alterations in prostate cancer treated with radiation therapy. *Urology* **51**: 346–351

Johnson MI, Robinson MC, Marsh C, Robson CN, Neal DE and Hamdy FC (1998) Expression of Bcl-2, Bax, and p53 in high-grade prostatic intraepithelial neoplasia and localized prostate cancer: relationship with apoptosis and proliferation. *Prostate* **37**: 223–229

Konishi N, Hiasa Y, Tsuzuki T, Tao M, Enomoto T and Miller GJ (1997) Comparison of *ras* activation in prostate carcinoma in Japanese and American men. *Prostate* **30**: 53–57

Krajewska M, Krajewski S, Epstein JI, Shabaik A, Sauvageot J, Song K, Kitada S and Reed JC (1996) Immunohistochemical analysis of bcl-2, bax, bcl-X, and mcl-1 expression in prostate cancers. *Am J Pathol* **148**: 1567–1576

Kuczyk MA, Serth J, Bokemeyer C, Machtens S, Minssen A, Bathke W, Hartmann J and Jonas U (1998) The prognostic value of p53 for long-term and recurrence-free survival following radical prostatectomy. *Eur J Cancer* **34**: 679–686

Masuda M, Takano Y, Iki M, Asakura T, Hashiba T, Noguchi S and Hosaka M (1998) Prognostic significance of Ki-67, p53, and Bcl-2 expression in prostate cancer patients with lymph node metastases: a retrospective immunohistochemical analysis. *Pathol Int* **48**: 41–46

Miyashita T and Reed JC (1995) Tumor suppressor p53 is a direct transcriptional activator of the human bax gene. *Cell* **80**: 293–295

Miyashita T, Harigai M, Hanada M and Reed JC (1994) Identification of a p53-dependent negative response element in the bcl-2 gene. *Cancer Res* **54**: 3131–3135

Moss SF, Scholes JV and Holt PR (1996) Abnormalities of epithelial apoptosis in multistep colorectal neoplasia demonstrated by terminal deoxyuridine nick end labeling. *Dig Dis Sci* **41**: 2238–2247

Moul JW, Friedrichs PA, Lance RS, Theune SM and Chang EH (1992) Infrequent *RAS* oncogene mutations in human prostate cancer. *Prostate* **20**: 327–338

Ogura E, Senzaki H, Yammamoto D, Yoshida R, Takada H, Hioki K and Tsubura A (1999) Prognostic significance of Bcl-2, Bcl-xL/s, Bax and Bak expressions in colorectal carcinomas. *Oncol Rep* **6**: 365–369

Oliveira C, Seruca R, Seixas M and Sobrinho-Simoes M (1998) The clinicopathological features of gastric carcinomas with microsatellite instability may be mediated by mutations of different "target genes": a

*© 2000 Cancer Research Campaign*

study of the TGFbeta RII, IGFII R, and BAX genes. *Am J Pathol* **153**: 1211–1219

Orita M, Iwahana H, Kanazawa H, Hayashi K and Sekiya T (1989) Detection of polymorphisms of human DNA by gel electrophoresis as single-strand conformation polymorphisms. *Proc Natl Acad Sci USA* **86**: 2766–2770

Shiraishi T, Muneyuki T, Fukutome K, Ito H, Kotake T, Watanabe M and Yatani R (1998) Mutations of *ras* genes are relatively frequent in Japanese prostate cancers: pointing to genetic differences between populations. *Anticancer Res* **18**: 2789–2792

Shurbaji MS, Kalbfleisch JH and Thurmond TS (1995) Immunohistochemical detection of p53 protein as a prognostic indicator in prostate cancer. *Hum Pathol* **26**: 106–109

Soong R and Iacopetta BJ (1997) A rapid and nonisotopic method for the screening and sequencing of p53 gene mutations in formalin-fixed, paraffin-embedded tumors. *Mod Pathol* **10**: 252–258

Smith DR, Ji CY and Goh HS (1996) Prognostic significance of p53 overexpression and mutation in colorectal adenocarcinomas. *Br J Cancer* **74**: 216–223

Stapleton AMF, Timme TL, Gousse AE, Li QF, Tobon AA, Kattan MW, Slawin KM, Wheeler TM, Scardino PT and Thompson TC (1997) Primary human prostate cancer cells harboring p53 mutations are clonally expanded in metastases. *Clin Cancer Res* **3**: 1389–1397

Theodorescu D, Broder SR, Boyd JC, Mills SE and Frierson HF Jr (1997) p53, bcl-2 and retinoblastoma proteins as long-term prognostic markers in localized carcinoma of the prostate. *J Urol* **158**: 131–137

Thibodeau SN, Bren G and Schaid D (1993) Microsatellite instability in cancer of the proximal colon. *Science* **260**: 816–819

Thomas DJ, Robinson M, King P, Hasan T, Charlton R, Martin J, Carr TW and Neal DE (1993) p53 expression and clinical outcome in prostate cancer. *Br J Urol* **72**: 778–781

Uchida T, Wada C, Shitara T, Egawa S and Koshiba K (1993) Infrequent involvement of p53 gene mutations in the tumorigenesis of Japanese prostate cancer. *Br J Cancer* **68**: 751–755

Watanabe M, Ushijima T, Kakiuchi H, Shiraishi T, Yatani R, Shimazaki J, Kotake T, Sugimura T and Nagao M (1994) p53 gene mutations in human prostate cancers in Japan: different mutation spectra between Japan and western countries. *Jpn J Cancer Res* **85**: 904–910

Wolter KG, Hsu YT, Smith CL, Nechushtan A, Xi XG and Youle RJ (1997) Movement of Bax from the cytosol to mitochondria during apoptosis. *J Cell Biol* **139**: 1281–1292

Yagi OK, Akiyama Y, Nomizu T, Iwama T, Endo M and Yuasa Y (1998) Proapoptotic gene BAX is frequently mutated in hereditary nonpolyposis colorectal cancers but not in adenomas. *Gastroenterology* **114**: 268–274

*© 2000 Cancer Research Campaign*
